# Exploring Cefiderocol Resistance Mechanisms in Burkholderia pseudomallei

**DOI:** 10.1128/aac.00171-23

**Published:** 2023-05-03

**Authors:** Carina M. Hall, Nawarat Somprasong, Johannah P. Hagen, Roxanne Nottingham, Jason W. Sahl, Jessica R. Webb, Mark Mayo, Bart J. Currie, Yuwana Podin, David M. Wagner, Paul Keim, Herbert P. Schweizer

**Affiliations:** a The Pathogen and Microbiome Institute, Northern Arizona University, Flagstaff, Arizona, USA; b Department of Biological Sciences, Northern Arizona University, Flagstaff, Arizona, USA; c Global and Tropical Health Division, Menzies School of Health Research, Charles Darwin University, Darwin, Northern Territory, Australia; d Department of Microbiology and Immunology at the Peter Doherty Institute for Infection and Immunity, University of Melbourne, Melbourne, Australia; e Institute of Health and Community Medicine, Universiti Malaysia Sarawak, Sarawak, Malaysia

**Keywords:** *Burkholderia pseudomallei*, cefiderocol, resistance

## Abstract

Cefiderocol is a siderophore cephalosporin designed mainly for treatment of infections caused by β-lactam and multidrug-resistant Gram-negative bacteria. Burkholderia pseudomallei clinical isolates are usually highly cefiderocol susceptible, with *in vitro* resistance found in a few isolates. Resistance in clinical B. pseudomallei isolates from Australia is caused by a hitherto uncharacterized mechanism. We show that, like in other Gram-negatives, the PiuA outer membrane receptor plays a major role in cefiderocol nonsusceptibility in isolates from Malaysia.

## TEXT

The initial treatment of melioidosis is with intravenous ceftazidime (CAZ) or meropenem (MEM), to which most primary isolates of B. pseudomallei are susceptible (1). Nevertheless, acquired resistance to both CAZ and MEM can evolve during therapy (1, 2). Cefiderocol (FDC) was designed for the treatment of β-lactam-resistant and multidrug-resistant (MDR) Gram-negative bacteria (3). It is a conjugate containing a cephalosporin moiety (combining structural components of CAZ and cefepime) and a siderophore (catechol) moiety (3, 4). The latter mediates FDC access into the periplasm via the outer membrane (OM) ferric siderophore receptor components of bacterial iron transport systems (5, 6). An Australian study showed that B. pseudomallei clinical isolates are highly susceptible *in vitro* to FDC with few resistant isolates (7). Of 246 clinical isolates tested, resistance to FDC was only observed in 3 isolates using CLSI clinical breakpoints for Acinetobacter baumannii and Pseudomonas aeruginosa (>4 μg/mL) or 4 isolates using the EUCAST clinical breakpoint for P. aeruginosa (>2 μg/mL) (7).

In the present study, we determined the FDC susceptibilities of 272 B. pseudomallei isolates from 16 countries with 16 isolates of unknown origin. Of the 272 strains, 160 (59%) were from Australia, 55 (20%) were from Thailand, and the rest were from 14 other countries spanning Asia/Southeast Asia, Africa, the Americas and Pacific, and Indian Ocean nations, and 16 isolates of unknown origin. FDC MICs were determined using broth microdilution (BMD) that was performed as previously described using 96-well plates provided by Shionogi & Co., Ltd. (Osaka, Japan) and prepared by International Health Management Associates (IHMA; Schaumburg, IL, USA) (7). Experiments with virulent B. pseudomallei were performed at BSL-3 in Select Agent-certified laboratory facilities at Northern Arizona University and employing compliant standard operating procedures approved by the Institutional Biosafety Committee. All isolates from this study were screened in biological duplicate with FDC concentrations of 0.03 μg/mL to 32 μg/mL. MICs were read at 16 to 20 h, except for 4 strains that were slow growers and required a 44-h incubation time. Iron-depleted cation-adjusted Mueller-Hinton broth (ID-CAMHB) was used according to the manufacturer’s recommendation (8). These analyses demonstrate that 9 of 272 B. pseudomallei isolates displayed either resistance (3 isolates) or nonsusceptibility (6 isolates) to FDC using the published CLSI breakpoints for A. baumannii (≤4 μg/mL susceptible; 8 μg/mL nonsusceptible; ≥16 μg/mL resistant) (Table 1) (9). In concordance with the previous Australian study, these results show that FDC resistance is rare but does exist in geographically diverse B. pseudomallei populations.

**TABLE 1:** B. pseudomallei clinical isolates with increased cefiderocol MIC

				MIC (μg/mL)^*a*^			
Strain	Country	Yr isolated	Sequence type	FDC	GEN	AZM	CAZ
1026b^*b*^	Thailand	1993	ST102	0.03	64	>64	≤4
MSHR1464	Australia	2003	ST131	32	>64	>64	<4
MSHR1713	Australia	2003	ST131	32	>64	>64	<4
MSHR7744	Australia	2013	ST131	8	8	>64	<4
MSHR5087	Malaysia	2011	ST881	8	<2	<4	<4
MSHR5089	Malaysia	2011	ST881	16	<2	<4	<4
MSHR5091	Malaysia	2011	ST881	8	<2	<4	<4
MSHR5093	Malaysia	2011	ST881	8	<2	<4	8
MSHR5095	Malaysia	2010	ST881	8	<2	<4	<4
MSHR5105	Malaysia	2011	ST881	8	<2	<4	<4

a Broth microdilution MICs determined with iron-depleted cation-adjusted Mueller-Hinton broth (FDC) or cation-adjusted Mueller-Hinton broth (AZM, GEN, CAZ). FDC, cefiderocol; GEN, gentamicin; AZM, azithromycin; CAZ, ceftazidime.

b Genome accession numbers: 1026b, GCF_000959125.1; MSHR1464, GCF_026315045.1*; MSHR1713, GCF_026315025.1*; MSHR7744, GCF_026315005.1*; MSHR5087, GCF_028201395.1*; MSHR5089, GCF_001980585.2*; MSHR5091, GCF_027946755.1*; MSHR5093, GCF_001980605.1; MSHR5095, SAMN14775583; MSHR5105, GCF_001980675.1. Asterisks mark deposited assemblies that are new as part of this work.

Although FDC is highly active against several problem pathogens, including A. baumannii and P. aeruginosa, resistance has been increasingly reported (10). Reduced susceptibility or resistance to FDC in clinical isolates of various pathogens has been attributed to diverse mechanisms, among which β-lactamases, siderophore receptors, and penicillin-binding protein 3 (PBP3) are frequently found, often acting in concert (10, 11). β-lactamases involved in FDC resistance include Pseudomonas extended resistant (PER) spectrum β-lactamases in A. baumannii (12, 13) and AmpC variants in P. aeruginosa, e.g., AmpC_E247K_ (14). An analysis of clinical A. baumannii isolates identified mutations in the *pbp3* gene encoding the main FDC PBP3 target (11, 13, 15). Laboratory experiments with P. aeruginosa and A. baumannii demonstrated that the PiuA OM catechol receptor, a component of a cognate TonB bacterial iron transport system, is involved in FDC uptake and resistance (16, 17). PiuA mutations have also been identified in clinical isolates of A. baumannii (11, 13) and P. aeruginosa in combination with an AmpC_L147F_ mutation (18). Since the mechanisms of FDC resistance in B. pseudomallei were unknown, our first objective in this study was to determine whether the bacterium possesses a PiuA homolog that is involved in FDC uptake and resistance.

**FIG 1:**
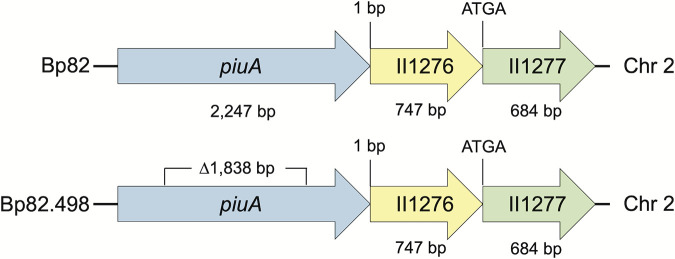
Genomic organization of B. pseudomallei *piuA*. The *piuA* gene is located on chromosome 2 where it seemingly forms an operon with two other genes, II1276 (BP1026B_II1276) encoding a hypothetical protein and II1277 (BP1026B_II1277) encoding a putative hydroxylase. Bp82 is an attenuated derivative of 1026b, hence the use of 1026b gene nomenclatures and sizes. Bp82.498 was derived by deletion of 1,838 bp DNA from within *piuA*.

Online protein BLAST analyses (https://blast.ncbi.nlm.nih.gov/Blast.cgi) using P. aeruginosa PAO1 PiuA (locus tag PA4514; www.pseudomonas.com) (19) as query identified a putative PiuA candidate in B. pseudomallei 1026b (BP1026B_II1275; *Burkholderia* Genome Database; www.burkholderia.com) (20). Protein sequence alignments and similarity predictions of selected PiuA protein sequences were performed using MUSCLE on the EMBL-EBI server (https://www.ebi.ac.uk/Tools/msa/muscle/) (21). These analyses revealed a 748-amino acid protein, which is 49.7% identical to P. aeruginosa PiuA. It is encoded by a 2,247-bp coding region on chromosome 2, whose product in the *Burkholderia* Genome Database is annotated as an OM ferric siderophore receptor (Fig. 1). This putative B. pseudomallei *piuA* gene was then deleted from the Select Agent excluded strain Bp82 (https://www.selectagents.gov) that is derived from the virulent 1026b (22), using published procedures (23) to arrive at the unmarked Δ*piuA* mutant Bp82.498 (Fig. 1). The presence of the desired deletion was verified by PCR amplification and Sanger sequencing. Bp82 and its parental strain 1026b exhibited an FDC susceptible phenotype (MIC ≤ 0.03 μg/mL) (Table 1), whereas the mutant, Bp82.498, had an MIC of 4 μg/mL, which was at least 128-fold higher than the Bp82 MIC.

Having demonstrated that deletion of PiuA substantially increases the FDC MIC in an engineered laboratory strain, we used BLAST to examine the genomes of the aforementioned 3 resistant and 6 nonsusceptible clinical B. pseudomallei isolates in comparison with strain 1026b. Three of these are genetically related isolates (2 resistant, 1 nonsusceptible) assigned to ST131 and obtained from a long-term chronic melioidosis infection of an Australian patient with bronchiectasis (24, 25) and the other 6 are also genetically related isolates (1 resistant, 5 nonsusceptible) assigned to ST881 and obtained from melioidosis patients from Malaysia (26). ST881 is a dominant clone seen in Sarawak, Malaysian Borneo and the 6 isolates were from 6 separate patients from 2 locations which are over 100 km apart. The isolates from Malaysia all contain 1 synonymous *piuA* single nucleotide polymorphism (SNP) and an 11-nucleotide TCGGAGAAGGC insertion between *piuA* nucleotides 1602 and 1603 that results in a frameshift causing expression of a truncated, nonfunctional, 633-amino acid PiuA. These results confirm that B. pseudomallei PiuA is involved in FDC uptake and resistance. However, rather than the frameshift, the isolates from Australia contain 5 synonymous SNPs in *piuA* that result in expression of the identical 748-amino acid PiuA observed in 1026b.

Examination of the CAZ, azithromycin (AZM), and gentamicin (GEN) MICs provided information about other possible FDC resistance mechanisms. All strains from Australia and Malaysia examined in this study are CAZ susceptible, which indicates that neither PenA β-lactamase overexpression (27, 28) or critical amino acid mutations (27, 29), nor PBP3 target mutations (30) contribute to their FDC resistance. For the nonresistant FDC isolates from Australia, lack of PenA and PBP3 involvement in FDC resistance was supported by sequence analyses. Both 1026b and the Australian isolates lacked the G to A mutation at position −78 in the *penA* upstream sequence that is required for increased *penA* transcription in acquired CAZ resistance (28). Compared to 1026b, the Australian isolates contain 1 synonymous and 1 nonsynonymous (C233T) SNP in *penA*. The nonsynonymous SNP causes the known S72F PenA mutation that results in increased amoxicillin + clavulanic resistance but not CAZ resistance (31, 32). A comparison of the *pbp3* gene of 1026b (30) and the Australian strains revealed 2 synonymous SNPs in *pbp3*. Lastly, it should be noted that the only slow growers in the 272 panel of global isolates that were tested for FDC susceptibility were 4 isolates from the same Australian patient (24, 25). Three of these isolates had elevated FDC MICs (8, 32, and 32 μg/mL) (Table 1), whereas one isolate exhibited a low MIC (0.06 μg/mL). These results indicate no direct relation between growth rate and FDC MIC.

The FDC-susceptible 1026b (MIC ≤ 0.03 μg/mL) and the FDC-resistant (MIC = 16 to 32 μg/mL) isolates from Australia, MSHR1464 and MSHR1713, are GEN and AZM resistant due to AmrAB-OprA efflux pump activity (33). The FDC susceptibility of 1026b expressing AmrAB-OprA indicates that FDC is not prone to efflux by this pump. This is comparable to P. aeruginosa, where expression of MexAB-OprM had no significant effect on FDC activity (16). It is of note that the FDC and GEN MICs in the more recent Australian strain MSHR7744 are different from the genetically related MSHR1464 and MSHR1713 isolates obtained 10 years earlier from the same patient. We do not yet understand the underpinnings for these observations, but they may be due to the genetic evolution that is well documented in B. pseudomallei isolates from this patient (24, 25). The 6 isolates from Malaysia are GEN and AZM susceptible because they are AmrAB-OprA deficient due to a nonsynonymous mutation within *amrB* (26).

In conclusion, our results confirm that the PiuA OM siderophore receptor plays a crucial role in B. pseudomallei’s FDC uptake and increased resistance. Clinical resistant isolates with MICs ≥16 μg/mL identified in a published study (7) and our studies presented here indicate the presence of additional resistance mechanisms that are not linked to resident β-lactamase and PBP3 expression. Further investigation will provide valuable insights into the possible application of FDC for the treatment of melioidosis.

### Data availability.

Genome assemblies that are new as part of this work were deposited to GenBank under accession numbers: GCF_026315045.1 (MSHR1464); GCF_026315025.1 (MSHR1713); GCF_026315005.1 (MSHR7744); GCF_028201395.1 (MSHR5087); GCF_001980585.2 (MSHR5089); and GCF_027946755.1 (MSHR5091).
